# The characteristics, occurrence, and toxicological effects of alternariol: a mycotoxin

**DOI:** 10.1007/s00204-024-03743-0

**Published:** 2024-04-25

**Authors:** Iman Saleh, Randa Zeidan, Mohammed Abu-Dieyeh

**Affiliations:** Biological Science Program, Department of Biological and Environmental Sciences, College of Art and Science, Qatar University, P.O. Box 2713, Doha, Qatar

**Keywords:** Alternariol, Mycotoxins, Health risk assessment, Mycotoxin occurance, Genotoxicity

## Abstract

*Alternaria* species are mycotoxin-producing fungi known to infect fresh produce and to cause their spoilage. Humans get exposed to fungal secondary metabolites known as mycotoxin via the ingestion of contaminated food. Alternariol (AOH) (C_14_H_10_O_5_) is an isocoumarins produced by different species of *Alternaria* including *Alternaria alternata*. AOH is often found in grain, fruits and fruits-based food products with high levels in legumes, nuts, and tomatoes. AOH was first discovered in 1953, and it is nowadays linked to esophagus cancer and endocrine disruption due to its similarity to estrogen. Although considered as an emerging mycotoxin with no regulated levels in food, AOH occurs in highly consumed dietary products and has been detected in various masked forms, which adds to its occurrence. Therefore, this comprehensive review was developed to give an overview on recent literature in the field of AOH. The current study summarizes published data on occurrence levels of AOH in different food products in the last ten years and evaluates those levels in comparison to recommended levels by the regulating entities. Such surveillance facilitates the work of health risk assessors and highlights commodities that are most in need of AOH levels regulation. In addition, the effects of AOH on cells and animal models were summarized in two tables; data include the last two-year literature studies. The review addresses also the main characteristics of AOH and the possible human exposure routes, the populations at risk, and the effect of anthropogenic activities on the widespread of the mycotoxin. The commonly used detection and control methods described in the latest literature are also discussed to guide future researchers to focus on mitigating mycotoxins contamination in the food industry. This review aims mainly to serve as a guideline on AOH for mycotoxin regulation developers and health risk assessors.

## Introduction

Mycotoxins-producing fungi belong to various fungal genera mainly *Penicillium*, *Fusarium*, *Aspergillus*, and *Alternaria* (Greeff-Laubscher et al. 2019). The genus *Alternaria* was originally described in 1816, with an increasing number of species being characterized since then (Ostry 2008). The major mycotoxin-producing *Alternaria* species is *Alternaria alternata*. Other mycotoxin-producing *Alternaria* species are: *Alternaria arborescens*, *Alternaria blumeae*,* Alternaria tenuissima*, *Alternaria tenuissima*, *Alternaria arborescens*, *Alternaria longipes*, *Alternaria radicina*, *Alternaria dauci*, *Alternaria infectoria *(Nan et al. 2022).

*Alternaria* species are traditionally classified based on the morphology of reproductive structures and sporulation patterns. Nowadays, molecular techniques are being used for fungal classification as a more reliable and less tedious method (Zhang et al. 2023). Some *Alternaria* fungi are saprophytic, which are usually found in outdoor environments and in/on surfaces like soil, wall papers, and textiles (Ostry 2008). However, most *Alternaria* species are plant pathogens that can adapt to various environmental conditions, including low humidity and low temperatures. Therefore, besides affecting plants during their growth stage, *Alternaria* may be a major causative agent of post-harvest diseases in fruits and vegetables during storage and transportation (Ji et al. 2021). *Alternaria* species cause worldwide economic losses by affecting a number of plants’ leaves, stems, flowers, and fruits. They are ranked as one of the highest loss-causing fungal genera among all plant pathogens (Behiry et al. 2022). Species belonging to *Alternaria* are necrotrophs, which can live on dead organic matter such as decaying wood and wood pulp, which allows them to survive for years in fields to infect future agricultural commodities (Chung 2012). They are also categorized as aeroallergens because of their light-weight spores can be dispersed by air (Grewling et al. 2020).

**Fig. 1:**
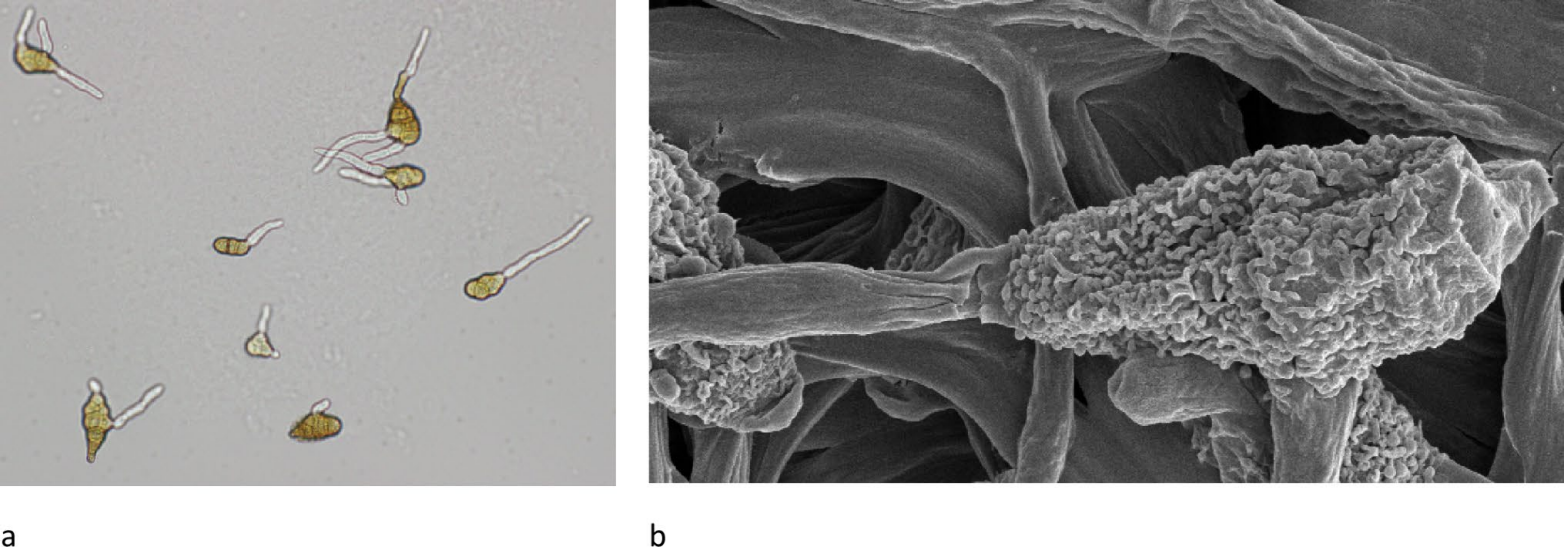
Microscopic images of *Alternaria alternata* germination tubes, **a** compound light microscope image (400×), **b** scanning electron microscope image (5000×)

*Alternaria* infection efficiency is enhanced by the melanized wall of its spores, which protects it from ultraviolet light and desiccation, and by the formation of multiple germ tubes per spore during germination (Fig. 1) (Chain 2011). *Alternaria* mycelium growth occurs at an optimum temperature of between 18 and 25 °C. However, spore infection and germination can occur within a wide temperature range of between 4 and 35 °C (Chain 2011). During infection, *Alternaria* species produce host-specific, and non-host-specific phytotoxins and extracellular enzymes to destroy plant cell walls at the infection site, which plays a major role in pathogenicity against plants (Wu and Wu 2019). Due to their tolerance to wide environmental conditions, *Alternaria* can infect a range of produce in various geographic locations, which cause the propagation of its mycotoxins (Louro et al. 2024).

Fruits and vegetables affected by *Alternaria* species show usually a visible rotten area, like the black mold on a tomato, to be avoided by consumers. In cereal grains, *Alternaria* cause a disease known as black point, which is characterized by the discoloration of the germ and seed. However, in some *Alternaria* diseases like the core rot of apples and black rot of citrus, the visible symptoms are only inside the plant, yet the mycotoxins would have diffused to all parts of the plant causing adverse health effects when consumed (Chain 2011; Pinto and Patriarca 2017). Fruit- and vegetable-based processed foods like jams and juices might contain levels of *Alternaria* mycotoxins due to the lack of industrial procedures to eliminate infected fresh produce prior to processing (Saleh and Goktepe 2019). Mycotoxin levels in fruit-based processed food also increases due to the lack of symptoms in fruits with *Alternaria* core infections (Patriarca 2019).

*Alternaria* toxins have received an increased research interest in the last few years, enabling the development of advanced and quick detection methods (Han et al. 2023a, b). Fungi belonging to the *Alternaria* genus produce more than 70 known mycotoxins belonging to three different structural groups: dibenzopyrone derivatives, perylene derivatives, and tetramic acid derivative (Pinto and Patriarca 2017). The most toxicologically concerning *Alternaria* toxins are: alternariol (AOH), alternariol monomethylether (AME), tenuazonic acid (TeA), tentoxin (TEN), altertoxin II (ATX II) and altenuene (ALT) (Babič et al. 2021; Schultz et al. 2022). Those mycotoxins were isolated and characterized between the years 1953 and 1986, with AOH first being discovered in 1953 (Ostry 2008). The most studied among *Alternaria* mycotoxins are the ones with benzopyrone groups which include the two major toxins: AOH and AME (Escrivá et al. 2017). Alternariol is often found in grains, fruits, and fruit-based food products such as jams and juices (Puvača et al. 2022). High levels of AOH have also been encountered in legumes, nuts, tomato and oilseed foods (Solhaug et al. 2016).

*Alternaria* mycotoxins can cause, like other mycotoxins, many adverse health effects in humans. In the last decade, scientists have proven in vitro* Alternaria* mycotoxins toxicity. Mutagenicity of *Alternaria* mycotoxin, in general, and the genotoxicity of AOH and AME, in particular, have been well demonstrated by showing DNA damage caused by indirect mechanisms (Aichinger et al. 2021). In addition, there is a correlation between the occurrence of *Alternaria* mycotoxins and esophageal cancer in the literature (Solhaug et al. 2016). Alternariol has also shown similarity to estrogen, which suggests a major endocrine disruptive role of AOH (Stiefel and Stintzing 2023).

Despite multiple studies proving the risks of *Alternaria* mycotoxins, worldwide regulation for these mycotoxins in food is still lacking. Exceptionally, the Bavarian health and food safety authority specified the tenuazonic acid limit in sorghum/millet-based infant food at 500 µg/kg content (Ji et al. 2023). In addition, the European Food Safety Authority (EFSA) performed a risk assessment for four of the known *Alternaria* mycotoxins (alternariol, alternariol monomethyl ether, tenuazonic acid, and tentoxin). As a result, thresholds for toxicological concern (TTC) levels of the four mycotoxins were set (EFSA 2016).

Several review articles have been published in the field over the past few years. However, most of them are related to the modes of detection of mycotoxins, to mycotoxins in specific commodities, or to *Alternaria* mycotoxins in general. Recent reviews focusing on AOH are lacking. The present review focuses on the characteristics of AOH, its environmental fate, its possible routes of exposure, its occurrence in different food products in the last decade, its toxicity on cells and animal models as occurring in the literature in the last two years, its carcinogenicity and anticancer activity as well as its possible control methods. This comprehensive review would serve as a guideline about AOH for mycotoxins regulating and policies developing entities, and for food scientists and health risk assessors around the world.

## Effect of anthropogenic activities on the spread of mycotoxins

Environmental factors including temperature and water activity are among the most significant factors in affecting mycotoxigenic fungi growth at pre-harvest and post-harvest levels (Gab-Allah et al. 2023). Anthropogenic activities including large-scale deforestation, the usage of fossil fuel as the main energy source, the over-exploitation of Earth’s resources, and other human activities have contributed to global climate change (Vagelas and Leontopoulos 2023). Concentrations of anthropogenic greenhouse gases (GHG) including methane, carbon dioxide, nitrous oxide, and chlorofluorocarbons have increased in the atmosphere in recent decades, resulting in global warming (Reineke and Schlömann 2023). Resulting climatic changes vary regionally. More frequent heat waves, extreme temperatures and precipitation events are expected in a number of regions. Yearly mean precipitation is expected to increase at high latitudes, many mid-latitude wet regions, and the equatorial Pacific; a decrease is anticipated in many mid-latitude and subtropical dry regions resulting in droughts (Medina et al. 2017).

Global warming and its associated changes in climate are likely to lead to an increased number of biotic and abiotic stresses on crops which would have variable effects on the interactions between crops and fungal pathogens such as mycotoxigenic fungi (Medina et al. 2017). Mycotoxins are climate-dependent, plant-related, and storage-associated problems. They are influenced by certain non-infectious factors such as the bioavailability of nutrients and insect damage, which in turn are driven by climatic conditions. Climate represents the key agro-ecosystem driving force of fungal contamination in agricultural commodities and therefore, in mycotoxin production (Paterson and Lima 2010).

An example of the effect of a climate change-related stress on the levels of fungal infections was observed on maize in northern Italy, between 2003 and 2004. Prolonged drought conditions and extreme elevated temperatures resulted in stressing maize plants, which made them more prone to fungal infections (Giorni et al. 2007). Quantitative estimations of the effects of global warming on mycotoxin contamination were conducted on Deoxynivalenol (DON) in wheat in northwestern Europe and on Aflatoxin B1 (AFB1) in maize and wheat in Europe. Results revealed the increase in contamination levels in both crops as a result of future climate (Medina et al. 2017).

In general, the increase in temperatures in areas with originally cool weather or temperate conditions might make those areas more liable to aflatoxins, Ochratoxin A, Patulin and other mycotoxins related to warm areas. Avoiding post-harvest diseases in such case would come with an increased cost (Tsitsigiannis et al. 2012). On the other hand, a possible positive effect of climate change is the excessive increase in temperatures in areas of the globe that are already hot, which might lead to the extinction of certain mycotoxin-producing fungi (Paterson and Lima 2010). Future changes in rainfall and temperature will modify the entire ecosystem. Modifications related to both the extinction and appearance of new insect and plant species would definitely affect the availability of fungal strains and therefore might bring novel mycotoxin threats to crops (Tsitsigiannis et al. 2012). Shifting geographic distribution of mycotoxigenic fungi in response to global warming will make them harder to control (Medina et al. 2017).

As an example of crops showing high levels of AOH contamination, storage of grains for instance would become more challenging with increased humidity, which might increase levels of AOH and other mycotoxins (Castañares et al. 2020). To avoid unexpected future problems that might cause unforeseen economic losses, a prediction system for possible mycotoxin levels could be developed. As weather forecasts have already become well developed to guide control strategies for various worldwide important diseases, it is similarly possible to relate weather-based plant disease forecasts to recent climate change models. We would, therefore, have an idea about the possible effects of environmental climate change in mycotoxins, including in their location, types, and extent of change (Paterson and Lima 2010).

Climate change is only one of the megatrends that cause long-term global effects. The European Environmental Agency (EEA) has set 11 global megatrends among which globalization, technological development and climate change have a major impact on fungal distribution around the world (Magyar et al. 2021). Globalization has facilitated the transfer of fungal spores overseas, as shown in a study conducted in Qatar on the fungal strains found growing on fresh produce in the domestic market including *Alternaria species*. Results showed that the country of origin is the most significant factor affecting the level of contamination and the type of fungi (Saleh and Al-Thani 2019).

The fungi detected on goods and packaging materials imported from different countries might infect local fresh produce and cause increases in mycotoxin levels and even lead to the introduction of new mycotoxins (Migliorini et al. 2015). The most common pathway for the movement of microorganisms across borders is through the trade of plants, especially potted living ornamental plants, where soil-borne microorganisms have a higher possibility of surviving transportation and becoming established at their destination. *Alternaria* is a common pathogen of plants’ green leaves which would increase the levels of mycotoxins worldwide (Santini et al. 2018). In the USA, annual plant imports increased between 1967 and 2010 by 500%. Similar trends were also observed in Europe and all over the world (Magyar et al. 2021). Nowadays, rapid transportation and reduced delivery times increase the survival of pathogens and lead to the spread of new species in new destinations. If an invasive fungus survives, adapts and multiplies in a new environment, its eradication becomes a great challenge. All of this adds to existing stresses and leads to unexpected mycotoxins in food products (Magyar et al. 2021).

Technological development is also one of the anthropogenic activities that affects mycotoxin distribution around the world. Fungi are well adapted to colonizing human-made material, which make their distribution vulnerable to technological development. For example, the effect of introducing new building materials may lead to the growth of unexpected fungi, depending on regional climate. It is important to study the interaction between fungi, substrates and climatic factors before introducing new technologies in construction (Magyar et al. 2021).

Finally, the increased application of chemical fungicides by farmers in agriculture has led to the emergence of multi-drug-resistant pathogens which are a public health concern (Saleh and Goktepe 2019). Development of biological controls that can limit fungal growth and, therefore, mycotoxin levels is a crucial research area to protect the environment from the adverse effects of chemicals and to combat multi-drug resistance strains (Saleh and Abu-Dieyeh 2021).

## Physical and chemical characteristics of AOH

**Fig. 2:**
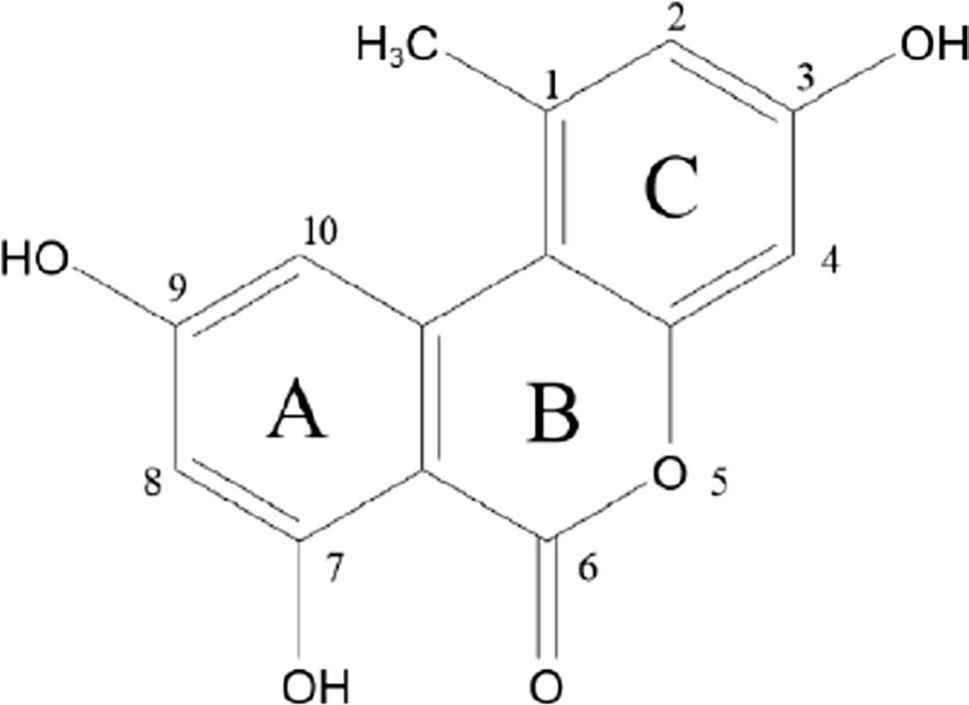
Alternariol chemical structure (PubChem 2022)

3,7,9-Trihydroxy-1-methyl-6H-dibenzo[b,d]pyran-6-one known as alternariol (AOH) (C_14_H_10_O_5_) is a benzochromenone belonging to the family of isocoumarins and its derivatives. AOH has a molar mass of 258.229 g/mole and it crystallizes from ethanol as colorless needles (PubChem 2022). The melting point of AOH is 350 °C. It is soluble in most organic solvents and it gives a purple color reaction with ethanolic ferric chloride (Chain 2011). The chemical structure of AOH is represented in Fig. 2.

## Alternariol (AOH) biosynthesis steps

Detailed knowledge of the biosynthesis of AOH and its metabolism is important to develop accurate detection methods and to better evaluate residual toxicological risks (Zhao et al. 2023).

*Alternaria alternata* produces more than 70 identified secondary metabolites, many of which are mycotoxins. Alternariol (AOH) and alternariol-9-methyl ether (AME) are two of the major food contaminants among *Alternaria* mycotoxins (Pinto and Patriarca 2017). However, the genetic-based biosynthesis of these two polyketide-based compounds is not well understood. One of the core enzyme categories involved in the biosynthesis of AOH and AME is polyketide synthases (PKSs) (Saha et al. 2012). Many of the biologically active fungal compounds are synthesized through polyketide biosynthesis pathways involving type I PKSs. Polyketide synthases are structurally and functionally similar to the mammalian fatty acid synthases (Cox and Simpson 2009).

Type I PKSs are made of large protein structures consisting of multiple covalently connected domains, which play a role in various catalytic steps. The basic type I PKS module consists of an acyltransferase (AT) domain, which is responsible for the starting stage of the polyketide synthesis. The elongation stages are the function of the domain acyl carrier protein (ACP) which connects the starter group to the keto-synthase (KS) domain, to catalyze carbon-bond formation. Elongation is terminated by the function of the domain thioesterase (TE) which hydrolyzes the completed polyketide chain from the ACP domain. Furthermore, many other functional domains can exist in the structure of type I PKS depending on their role. This includes keto-reductase (KR), dehydratase (DH), enoyl-reductase (ER), and methyl-transferase (MT) (Weissman 2020).

Saha et al. (2012) identified ten PKSs genes in the genome of *A. alternata*. Among the identified genes, two had their expression correlated with the production of AOH and AME (pksJ and pksH). The enzymes belong to type I-reducing polyketide synthases with 2222 and 2821 amino acid lengths, respectively (Saha et al. 2012).

**Fig. 3:**
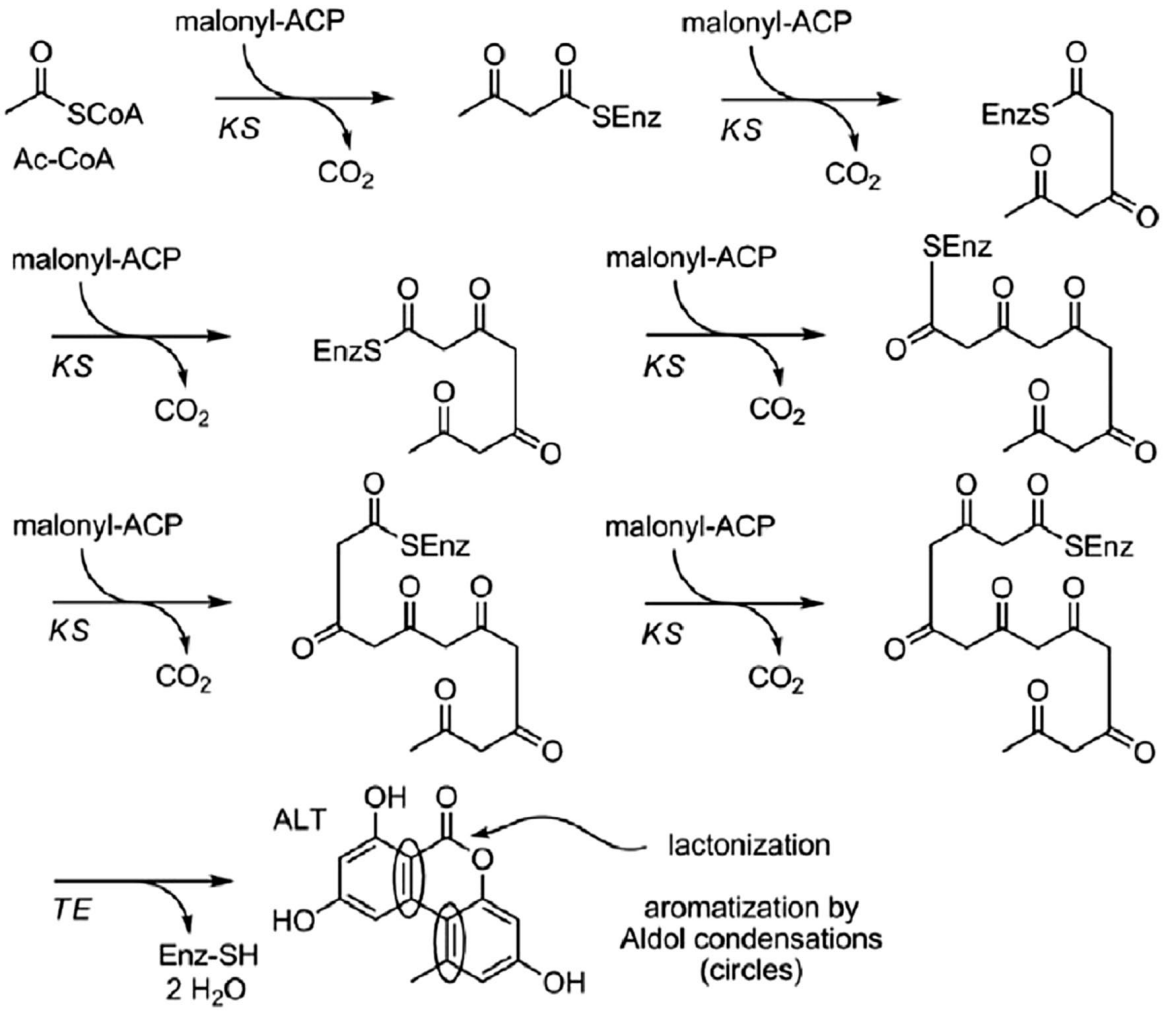
Biosynthetic suggested pathway for alternariol and alternariol-9-methyl ether (Saha et al. 2012)

Figure 3 represents a simple suggested model of AOH and its methylated derivative AME biosynthesis. In this model only ACP and KS domains are needed to initiate and elongate the polyketide, in addition to TE, to finalize it. In this model, biosynthesis starts with acetyl-CoA and consists of six condensation reactions, in each of which, activated malonate is integrated together with the loss of a carbonate group. As only two keto-synthase domains have been identified during alternariol biosynthesis, it could be likely that the six condensation reactions are catalyzed by the same domain. The aromatization process, which leads to the final natural product, could have happened before or after being liberated from the enzyme complex catalyzed by a thioesterase. Similarly, lactonization is possible either together with the liberation process or directly after it. Both steps (aromatization and lactonization) are likely to occur spontaneously without requiring enzymes (Saha et al. 2012).

It is worth mentioning, when describing AOH biosynthesis, that changes in the osmotic status of the substrate affect alternariol production. High environmental osmolarity is usually transmitted to the transcriptional level of downstream regulated genes by high osmolarity glycerol (HOG) signaling a cascade which is a MAP kinase transduction pathway. *Alternaria alternata* HOG gene (AaHOG) plays an important role in alternariol biosynthesis regulation (Graf et al. 2012).

## Alternariol (AOH) environmental fate

*Alternaria* toxins can be partially metabolized in plants to form a large number of conjugated metabolites. The toxicological relevance of modified mycotoxins forms and their occurrence in food is still largely unexplored. High-resolution mass spectrometry (HRMS) techniques are being developed to detect mycotoxins in their modified forms (Righetti et al. 2016). Mycotoxin bound to more polar substances such as glucose, amino acids and sulfates are known as masked mycotoxins, which are a health concern (Chain 2011). A study has demonstrated that AOH can conjugate well with glucose in cultured tobacco BY-2 cells, demonstrating that masked AOH can be directly formed in plant cells (Hildebrand et al. 2015). *Alternaria alternata* has also been shown to produce alongside AOH, a sulfate conjugate of the mycotoxin and sulfate/glucoside conjugate of AOH. Alternariol sulfate and AOH glucoside have been encountered in certain types of foods (Soukup et al. 2016; Walravens et al. 2016). Having free hydroxyl groups available for metabolic conjugation, AOH might occur in many masked forms including, alternariol-3-glucoside (AOH3G), alternariol-3-sulfate (AOH3S), alternariol monomethyl ether-3-glucoside (AME3G), and alternariol monomethyl ether-3-sulfate (AME3S) (Escrivá et al. 2017).

Alternariol can undergo aromatic hydroxylation by CYP450 enzymes and by the enzymes of the first phase of metabolism producing catechols and hydroquinones, which are involved in reactive oxygen species (ROS) generation, to cause cell toxicity. This supports the relevance of a possible in vivo oxidative metabolism of this mycotoxin (Burkhardt et al. 2011). At the same time, the presence of AOH increases transcription of CYP450 in cells (Aichinger et al. 2021). Knowledge about the toxicity of the AOH oxidative metabolites is crucial in assessing the health risks of mycotoxin.

Lower amounts of mycotoxins would be expected in processed foods, compared to fresh produce, provided that the processing steps deteriorate mycotoxin. In the case of AOH, a study conducted on the effect of baking in the level of mycotoxins in the final baked products (using spiked whole-meal wheat flour), showed that wet baking did not affect the level of AOH while dry baking caused a significant reduction in mycotoxin (Siegel et al. 2010). However, a long fermentation period showed a reduction in AOH in whole wheat dough preparation (Janić Hajnal et al. 2020).

*Alternaria* species are a common cause of moldy core diseases in many fruits including citrus fruits and apples. Infected fruits cannot be detected as they might not show any visible external symptoms and therefore might be destined for industrialization (Pavicich et al. 2020). A study conducted on clear and cloudy apple juices, to evaluate the efficacy of the different treatment steps in lowering AOH levels, showed that the casual clear juice treatment stages, including pectinolytic enzyme treatment and pasteurization, did not have any significant effect on the level of AOH found in raw juice. However, fining with subsequent filtration, using activated charcoal/bentonite lowered the AOH level 79µg/L to the limit of quantification (4.6 µg/L). As for the cloudy juice processing steps, no step, including centrifugation *or* pasteurization, showed any effect on the studied level of the mycotoxin (Aroud et al. 2021). Therefore, if the fruits used in juice production have a certain AOH level, their juices are likely to maintain that contamination unless special treatments such as ultra-filtration (clarification step) are applied (Pavicich et al. 2020). Similarly, a recent study has proven the detection of AOH and it conjugates in the final drink after malting and brewing during beer preparation, which indicates that the processing stages are not enough to eliminate mycotoxin, originating from contaminated raw barley and malt (Prusova et al. 2022).

*Alternaria* species are common in nature and they may affect in-field plants. A recent study has evaluated the levels of AOH in different parts of winter wheat plants by inoculating AOH into their nutrient solutions in hydroponic system to simulate soil contamination in the field. After one week of exposure, 5% of the inoculated AOH was recovered from the plants, with 58% in the roots, 16% in the crown, and 1% in the leaves. The recovered fraction increased to 21% of the inoculated amount after two weeks of exposure. Beside AOH recovery, 26 AOH conjugates were detected in different parts of the plants (Jaster-Keller et al. 2023). The study indicates that in-field contamination would lead to significant levels of mycotoxins and their masked forms in fresh produce, even without actual fungal contamination of a growing plant.

Masked mycotoxins are mycotoxins associated with other molecules by covalent or non-covalent bonds, which allow them to escape the usual mycotoxin detection methods due to differences in polarity between the native mycotoxin and their metabolites. Since it is possible that a masked mycotoxin rereleases its native toxic form after enzymatic hydrolysis in the human digestive tract, human exposure levels to AOH can be higher than estimated. Very limited data is available on the occurrence of mycotoxin metabolites in food or animal feed (Escrivá et al. 2017). On the other hand, some recent studies demonstrate a decrease in *Alternaria* mycotoxins in general and AOH in particular in the digestive track. An in vitro short-term fecal incubation assay showed a reduction in mycotoxin concentrations. Additionally, DNA strand breaks usually induced by *Alternaria* mycotoxins were significantly quenched by the end of the 3h incubation period, while some other genotoxicity mechanisms were not affected. Ingested mycotoxins might interact with the gut microbiota and food constituents, which would modify their bioavailability and overall toxicity. Although results did not show a direct correlation between the metabolic activity of the gut microbiota and modifications in mycotoxin content, it is possible that mycotoxins were adsorbed into bacteria cells and into food constituents, which would lower their presence and their genotoxicity. Additional studies are needed to understand the fate of AOH in the digestive system (Crudo et al. 2020).

## Alternariol exposure routes

Humans and animals can get exposed to mycotoxins via the consumption of contaminated food products, including fruits and vegetables in their fresh and processed forms (El-Sayed et al. 2022). Fungal diseases can occur in-field through contaminated soil, air and irrigated water (Jain et al. 2019). They can also affect fresh produce at different post-harvest levels. Moreover, worker or harvesting equipment can also serve as a contamination source if hygienic practices are not strictly followed (Chatterjee et al. 2023). Infectious fungi might also affect fruits and vegetables during transportation or storage via contaminated containers. As *Alternaria species* can grow at low temperatures, they can infect produce during refrigerated transportation or storage (Li et al. 2023). At storage and display levels, cross-contamination becomes a major concern. Final food processing steps might also lead to fungal contamination (Saleh and Goktepe 2019). All of this can lead to mycotoxin-contaminated fresh produce, which is a major risk factor on human health (El-Sayed et al. 2022).

## Tolerable levels of alternariol (AOH)

There are no regulations for AOH levels in food up to now despite its known toxicity (Ji et al. 2023). Alternariol is still within food contaminants called “emerging mycotoxin” (Aichinger et al. 2021). According to the European Food Safety Authority (EFSA) the threshold for toxicological concern (TTC) of AOH is 2.5 ng/Kg bw/day (Solhaug et al. 2016). Nevertheless, the nature of genotoxicity of AOH is not fully understood. The fact that AOH can be metabolized into DNA adducts indicates that even low absorbed amounts of mycotoxin are concerning (Aichinger et al. 2021).

The highest recent human exposure rate to AOH, according to EFSA is in toddlers, with a mean exposure of between 3.8 and 71.6 ng/kg bw/day (EFSA 2016). This number is higher than the TTC for potential genotoxic substances, recently referred to as potential DNA-reactive mutagens, of 2.5 ng/Kg bw /day (EFSA 2019).

Indicative levels for AOH are set in certain foods by the European Union (EU). Set levels are based on the EFSA database. Samples with contamination levels above indicative levels require further investigations to limit the factors leading to the presence of AOH, such as initial fresh produce contamination or elevated mycotoxins levels caused by food processing. However, indicative levels are not food safety levels. Alternariol indicative levels in cereal-based foods for infants and young children is as low as 2 µg/Kg. At the same time, 10 µg/Kg is the indicative level for processed tomato products and sunflower oil, while 30 µg/Kg is the indicative level for sesame and sunflower seeds (EU 2022).

In addition, it is not fully understood if AOH in the masked-mycotoxin form can be hydrolyzed and absorbed in the gastrointestinal tract, which therefore adds to the overall exposure rate.

## Populations at risk of AOH exposure

Alternaria mycotoxins in general and AOH in particular occur at high levels in fruits, fruit-based food products, vegetables, cereal-based food products, tomatoes, and tomato-based food products. Populations with diets based on these foods categories are the most exposed to AOH. This includes infants and toddlers. In addition, vegetarians are generally more exposed to mycotoxins and, therefore, to AOH than the general population (EFSA 2016).

Analysis conducted on contamination levels in food products and mean consumption data of those food products showed possible exposure levels to different mycotoxins. A study conducted on tomato products, baked products, sunflower seeds, fruit juices and vegetable oils showed that, based on consumption rates of the population studied, the average daily exposure to AOH might reach 1400% of the suggested EFSA TTC level (Hickert et al. 2016).

## Alternariol contamination levels in food products

The main route of exposure to mycotoxins is the direct consumption of contaminated food products (Saleh and Goktepe 2019). Prolonged exposure to AOH has adverse effects on human health (El-Sayed et al. 2022). Advanced analytical methods for mycotoxin detection from fresh produce and food-based products are crucial in determining contamination levels and therefore in setting appropriate toxicological standards. The determination of *Alternaria* mycotoxins is largely based on a sequence of steps, starting with the pre-treatment of samples, followed by clean-up through solvent partitioning or solid phase extraction (Gab-Allah et al. 2023). Solid–liquid extraction with acetonitrile or ethyl acetate is the most common extraction method (Escrivá et al. 2017). The final separation and detection of mycotoxins occur through different methods, including chromatographic techniques (thin layer chromatography; high-performance liquid chromatography (HPLC); liquid chromatography–mass spectrometry (LC–MS); gas chromatography–mass spectrometry (GC–MS) and others), immunological techniques (enzyme-linked immunosorbent assay (ELISA); lateral flow immune-chromatographic assay (LFIA); fluorescence polarization immunoassay (FPIA) and others), biosensors techniques, and some sophisticated methods such as near-infrared spectroscopy (NMR) and others (Gab-Allah et al. 2023).

**Fig. 4:**
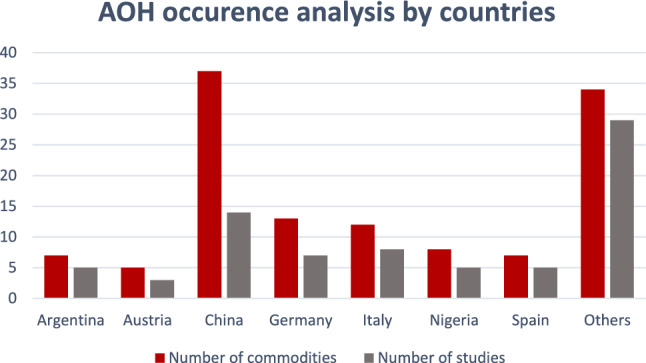
Number of commodities showing AOH occurrence and the number of studies from which the data was extracted, per country

Worldwide, multiple studies have surveilled the levels of AOH in fruits, vegetables and derived products, mainly in tomatoes, apples, cereals, and cereals by-products (Escrivá et al. 2017). Stability of mycotoxins during food processing is a major factor that adds to a mycotoxin’s significance as a risk factor (Avîrvarei et al. 2023). Being available in cereals, stability of *Alternaria* mycotoxins was evaluated during wet and dry baking, most of *Alternaria* toxins were stable during wet baking while significant degradation occurs during dry baking with AME and AOH being the most stable (Siegel et al. 2010). Alternariol showed heat stability up to 100 °C in sunflower flour (Lee et al. 2015). Stability of AOH has also been evaluated in beverages such as apple juice and wine to show stability up to five weeks in spiked apple juice and up to eight days in spiked white wine at room temperature (Fernández-Cruz et al. 2010). The stability shown, highlights the importance of AOH surveillance analyses in food products. Considering the possibility of co-occurrence of multiple mycotoxins in food products makes the presence of even trace amounts of a particular mycotoxin significant (Muñoz-Solano and González-Peñas 2023). Table 1 summarizes the contamination levels of AOH in food products as reported by studies conducted in the last ten years. Levels of AOH were recorded in 127 commodities belonging to different food categories including beverages, fresh and dried fruits and vegetables, nuts, cereals, processed foods, and other food products. As an emerging mycotoxin, studies reporting AOH occurrence levels have started to increase in number in the last five years. This can be inferred from the number of articles appearing in the database search per year. Studies related to AOH occurrence are mainly conducted in China and in some European counties (Fig. 4), around 26% of the data covered in Table 1 is reported from China.

**Table 1:** Contamination levels of AOH in food samples, comprehensive data for the last decade

Food category	Sample type	Country	*n*^a^	*P*^b^	Contamination levels range (μg/L or µg/Kg)^d^	Average contamination level (SD) (μg/L or µg/Kg)^d^	References
Beverages	Beer	Italy	30	100	6.04–23.2	–	(Prelle et al. 2013)
	Beer (maize)	Cameroon	14	21	0.05–0.6	0.30 ± 0.1	(Abia et al. 2013)
	Wine	Netherland	62	4.8	6.4–12	–	(Pizzutti et al. 2014)
	Apple juice	China	15	46.7	0.1–7.94	–	(Fan et al. 2016)
	Apple juice	Germany	20	15	2.1–4.31	3.52 ± 1.1	(Zwickel et al. 2016)
	Beer	Germany	44	100	0.23–1.6	0.56 ± 0.29	(Bauer et al. 2016)
	Fruits juices	Germany	23	56.5	0.65–16	3.10 ± 0.7	(Hickert et al. 2016)
	Grapes juice	Germany	8	88	1.58–6.45	4.01 ± 1.2	(Zwickel et al. 2016)
	Multi-fruits juice	Germany	13	15	1.78–6.21	-	(Zwickel et al. 2016)
	Red wine	Germany	14	93	1.68–7.65	3.30 ± 1.1	(Zwickel et al. 2016)
	Tomato juice	Belgium	28	71	<27	2.10 ± 0.1	(Walravens et al. 2016)
	White wine	Germany	11	36	0.65–1.19	0.95 ± 0.12	(Zwickel et al. 2016)
	Wine	China	12	91.6	0.04–0.7	-	(Fan et al. 2016)
	Berry juice	Spain	32	34	2.5–85.1	18.70 ± 1	(Juan et al. 2017)
	Red wine	Thailand	100	12	2.25–7.97	5.28 ± 0.12	(Puangkham et al. 2017)
	Beer (maize based)	South Africa	32	69	<54	47.00 ± 0.12	(Adekoya et al. 2018)
	Apple juice	Spain	80	29	<1213	207.00 ± 12	(Pallarés et al. 2019)
	Beer	Spain	40	90	2.01–49.82	19.39 ± 13	(Carballo et al. 2021)
	Green tea infusion	Morocco	11	40	<5.9	2.60 ± 1.3	(El Jai et al. 2021a, b)
	Beer	Czech republic	5	100	1.2	0.30–2.7	(Prusova et al. 2022)
	Apple juice	Argentina	15	26.7	2.2–6.2	4.50 ± 1.6	(Pavicich et al. 2023)
Fresh fruits and vegetables	Strawberry	Spain	24	66.7	8–752	103.20 ± 1	(Juan et al. 2016a, b)
	Apple	Netherland	11	9	<2	–	(López et al. 2016)
	Capsicum	Argentina	48	21	3–98	29.00 ± 1	(da Cruz Cabral et al. 2016)
	Cherry	China	55	5.5	0.16–1.44	0.87	(Qiao et al. 2018)
	Tomatoes (organic)	Spain	48	35	95.4–1,318.6	386.80 ± 7	(Estiarte et al. 2018)
	Tomatoes (non-organic)	Spain	34	21	336.5–1,436.9	1000.00 ± 150	(Estiarte et al. 2018)
	Apple	China	205	27.8	6.71–8517	935.96 ± 178.37	(Li et al. 2020)
	Apple	China	13	100	0.4–585.4	–	(Tang et al. 2020)
	Green Coffee	Switzerland	78	3.8	<1.7	–	(Mujahid et al. 2020)
	Jujube	China	25	56	<574.51	82.58 ± 1	(Fan et al. 2022)
	Apple	Italy	10	60	0.8–599.1	159.90 ± 6.92	(Carbonell-Rozas et al. 2023)
	Grapefruits & Pummelo	China	17	52.9	1.1–9.5	5 ± 1	(Han et al. 2023a, b)
	Lemons	China	31	64.5	1.9–62.4	8.7 ± 1	(Han et al. 2023a, b)
	Oranges	China	100	60	1.1–27.7	7.4 ± 1	(Han et al. 2023a, b)
	Tangerine	China	33	63.6	1.4–23.9	9.3 ± 1	(Han et al. 2023a, b)
Dried fruits	Raisin	China	57	5.3	3.5–15.6	8.90 ± 0.7	(Wei et al. 2017)
	Dried wolfberries	China	54	3.7	5.9–27.4	16.60 ± 0.9	(Wei et al. 2017)
	Dried capsicum	Italy	23	43	<428.4	78.00 ± 7	(Gambacorta et al. 2018)
	Dried figs	China	20	20	0.7–10.9	5.6 ± 0.11	(Wang et al. 2018)
	Dried jujubes	China	35	5.7	10.4–17.3	13.7 ± 0.22	(Wang et al. 2018)
	Raisin	China	30	6.7	8.0–11.2	9.8 ± 0.3	(Wang et al. 2018)
Tuberous crop	Potato	China	6	16.7	–	3.01 ± 1	(Ji et al. 2023)
	Sweet potato	China	6	33.3	–	2.74 ± 1	(Ji et al. 2023)
	Cassava	China	6	33.3	–	2.79 ± 1	(Ji et al. 2023)
Legume	Soybeans	Argentina	50	16	25–211	–	(Oviedo et al. 2012)
	Legumes and nuts	Italy	27	18	16–46	31.50 ± 0.7	(Lattanzio et al. 2022)
Nuts	Hazelnuts	Austria	22	90	<650	78.00 ± 2.0	(Varga et al. 2013)
	Peanuts	Austria	15	20	–	4.40 ± 0.7	(Varga et al. 2013)
	Nuts	China	133	14.3	1.4–142.9	28.6 ± 0.67	(Wang et al. 2018)
	Almond	Italy	17	12	0.34–0.37	0.35 ± 0.1	(Narváez et al. 2020)
	Pistachio	Italy	15	6	–	7.75 ± 0.1	(Narváez et al. 2020)
	Walnuts	Italy	22	53	0.29–1.65	0.67 ± 0.2	(Narváez et al. 2020)
Cereals	Wheat	Germany	1064	8.1	<831.7	–	(Müller and Korn 2013)
	Finger millet	Ethiopia	34	27.3	<104	10.00 ± 1	(Chala et al. 2014)
	Maize	Nigeria	70	18.6	0.8–57	10.00 ± 16	(Adetunji et al. 2014)
	Maize	Denmark	82	2.4	<24	18.00 ± 6	(Storm et al. 2014)
	Sorghum	Ethiopia	70	58.6	<70.62	18.00 ± 1	(Chala et al. 2014)
	Wheat	Serbia	92	12	<48.9	18.60 ± 17.3	(Janić Hajnal et al. 2016)
	Wheat	Italy	74	31	8–121	11.00 ± 1	(Juan et al. 2016a, b)
	Wheat	China	370	47	1.3–74.4	12.90 ± 1	(Xu et al. 2016)
	Maize	Brazil	148	3.3	<9.4	0.16 ± 0.1	(Oliveira et al. 2017)
	Sesame	Nigeria	24	21	1.14–3.5	–	(Toba 2018)
	Sorghum	Africa (sub-Saharan countries)	1533	2.5	75–1090	212 ± 203	(Ssepuuya et al. 2018)
	Maize	Nigeria	12	100	0.6–0.95	0.73 ± 0.3	(Oyeka et al. 2019)
	Wheat	Canada	30	6.6	4–8	–	(Tittlemier et al. 2019)
	Barley	Argentina	26	80.7	391–1689	623.00 ± 5	(Castañares et al. 2020)
	Barley	Argentina	34	47	368–1254	801.00 ± 17.3	(Castañares et al. 2020)
	Oat groats	Canada	14	71	4–50	28.00 ± 1	(Tittlemier et al. 2020)
	Oat hulls	Canada	14	71	4–305	25.00 ± 1	(Tittlemier et al. 2020)
	Barley	Russia (Urals, West Serbia)	49	12	2–8	–	(Orina et al. 2021)
	Barley	Slovenia	107	4	<44	31.00 ± 1	(Babič et al. 2021)
	Oat	Russia (Urals, West Serbia)	13	46	4–53	18.00 ± 5	(Orina et al. 2021)
	Oat	Slovenia	15	33	<1289	514.00 ± 11	(Babič et al. 2021)
	Triticale	Slovenia	81	10	<150	58.00 ± 1	(Babič et al. 2021)
	Rye	Slovenia	31	39	<116	75.00 ± 1	(Babič et al. 2021)
	Spelt	Slovenia	18	28	<1836	569.00 ± 11	(Babič et al. 2021)
	Wheat	Canada	323	74	–	2.20 ± 3.3	(Limay-Rios and Schaafsma 2021)
	Wheat	Russia (Urals, West Serbia)	116	31	2–26	–	(Orina et al. 2021)
	Wheat	Slovenia	181	8	<69	39.00 ± 1	(Babič et al. 2021)
	Maize	Cambodia, Laos, Myanmar, and Thailand	125	70	0.03–3.5	0.10 ± 0.1	(Siri-anusornsak et al. 2022)
	Rice bran	Cambodia, Laos, Myanmar, and Thailand	125	40	0.03–0.8	0.10 ± 0.1	(Siri-anusornsak et al. 2022)
	Sorghum	Ethiopia	47	58.7	0.73–46.4	3.39 ± 0.9	(Mohammed et al. 2022; Nagda and Meena 2024)
	Wheat	Serbia	40	10	2.1–5.3	3.30 ± 1.3	(Puvača et al. 2022)
	Wheat (durum)	Italy	70	55.7	<25.2	9.70 ± 4.5	(Senatore et al. 2023)
	Wheat (durum)	Slovakia and Germany	40	60	2.27–19.87	6.96 ± 1	(Wachowska et al. 2023)
Processed food products	Groundnut soup	Cameroon	15	47	0.04–3	0.70 ± 0.9	(Abia et al. 2013)
	Tomato-based products	Italy	10	50	4–6.8	–	(Prelle et al. 2013)
	Dried noodles	China	52	5.8	9.59–11.8	10.70 ± 1.09	(Zhao et al. 2015)
	Wheat flour	China	181	6.1	16.0–98.7	30.20 ± 23.6	(Zhao et al. 2015)
	Tomato-based products	Germany	34	70.6	6.1–25	13.00 ± 0.9	(Hickert et al. 2016)
	Tomato concentrate	Belgium	27	85	<31	7.60 ± 1	(Walravens et al. 2016)
	Tomato sauce	Netherland	8	50	2–25	–	(López et al. 2016)
	Tomato sauce	Belgium	28	86	<41.6	2.70 ± 0.1	(Walravens et al. 2016)
	Vegetable oils	Germany	19	47.3	–	6.00 ± 0.4	(Hickert et al. 2016)
	Cherry jam	China	13	100	<2.2	0.67 ± 0.1	(Qiao et al. 2018)
	Cherry dried	China	12	92	0.34–2.22	2.06 ± 0.1	(Qiao et al. 2018)
	Family Cereal	Nigeria	26	15.4	0.4–0.9	0.60 ± 0.2	(Ojuri et al. 2018)
	Infant formula	Nigeria	17	5.9	–	0.70 ± 0.01	(Ojuri et al. 2018)
	Tom bran	Nigeria	30	30	1.2–7.2	2.10 ± 1.9	(Ojuri et al. 2018)
	Tomato concentrate	Spain	30	47	22.6–137.4	45.00 ± 2.9	(Estiarte et al. 2018)
	Tomato sauce	Austria	56	30	1.2–20.8	6.70 ± 4.3	(Puntscher et al. 2019)
	Cereal-based baby foods	Germany	19	37	<7.17	0.89 ± 0.2	(Gotthardt et al. 2019)
	Infants food (homemade)	Nigeria	53	18.9	0.4–7.2	1.90 ± 1.1	(Ojuri et al. 2019)
	Infants food (commercial)	Nigeria	84	6	0.4–0.9	0.60 ± 0.2	(Ojuri et al. 2019)
	Wheat flour	Austria	100	13	<13	3.50 ± 3.3	(Puntscher et al. 2019)
	Vegetable oils	India	100	34	5.18–938.3	89.37 ± 5	(Bansal et al. 2021)
	Biscuit	China	294	3.1	<3.07	0.21 ± 0.1	(Ji et al. 2022a)
	Coffee	Tunisia	100	33	76.8–348.7	228.40 ± 116	(Oueslati et al. 2022)
	Common cereals	China	216	17.6	<45.2	2.02 ± 0.5	(Ji et al. 2022a)
	Infant cracker	China	277	3.2	<3.1	0.20 ± 0.1	(Ji et al. 2022a, b)
	Infant noodle	China	229	7.9	<3.2	0.30 ± 0.1	(Ji et al. 2022a, b)
	Millet	China	69	15.9	<3.5	0.40 ± 0.1	(Ji et al. 2022a, b)
	Mixed grain cereals	Germany	13	85	0.4–2	0.90 ± 0.6	(Rehagel et al. 2022)
	Wheat floor	China	54	16.7	<45.2	3.30 ± 1	(Ji et al. 2022a, b)
	Apple infant food	Argentina	20	35	1.7–13.7	5.00 ± 4.4	(Pavicich et al. 2023)
	Apple puree	Italy	20	60	2.1–45.6	10.30 ± 1.75	(Carbonell-Rozas et al. 2023)
	Tomato based food	Argentina	79	13	4.7–105.3	40.7 ± 2	(Maldonado Haro et al. 2023)
	Tomato Sauces	China	60	46.7	1.1–24.6	6.70 ± 1	(Xu et al. 2023a, b)
	Tomato Ketchup	China	38	52.6	1.1–27.2	6.70 ± 1	(Xu et al. 2023a, b)
	Tomatoes chopped	China	26	42.3	1.7–12.8	5.30 ± 1	(Xu et al. 2023a, b)
	Tomato hotpot additives	China	24	75	1.1–17.7	6.40 ± 1	(Xu et al. 2023a, b)
	Tomato juices	China	13	46.2	1.9–7.9	5.00 ± 1	(Xu et al. 2023a, b)a
Others	Sunflower seeds	Germany	11	54.5	16–39	27.00 ± 2.3	(Hickert et al. 2016)
	Sunflower seeds	South Africa	159	8.2	4.6–246	62.80 ± 2.5	(Hickert et al. 2017)
	Sunflower seeds	Austria	39	31	0.7–2.9	1.40 ± 0.6	(Puntscher et al. 2019)
	Mixed spices	Lebanon	94	40	<636.4	45.00 ± 1.7	(Gambacorta et al. 2019)
	Mixed herbs	Lebanon	37	19	<64.2	9.70 ± 1	(Gambacorta et al. 2019)
	Aromatic and medicinal plants	Morocco	40	85	2.3–309.5	126.20 ± 40.4	(El Jai et al. 2021b)
	Green tea	Morocco	111	40	1.7–5.9	2.60 ± 1.3	(El Jai et al. 2021a, b)
	Olive oil	China	20	25	<7.53	–	(Lin et al. 2022)
	Chili powder	China	27	3.7	10.4	10.40 ± 1	(He et al. 2023)
	Plant-based meat alternatives	Italy	13	23	5.6–12.1	9.60 ± 1.5	(Augustin Mihalache et al. 2023)

^a^Number of samples

^b^Percentage of contaminated samples

^c^Limit of quantification

^d^Levels are measured in liquid samples in μg/L and in solid samples in µg/Kg

Among the four records of AOH levels in apples, the highest level was indicated in samples collected in China, with an AOH average level of 935.96 ± 178.37 µg/Kg, followed by samples from Italy, with an average occurrence level of 159.90 ± 6.92 µg/Kg. As for apple juice samples, five records were included, with the highest average level in samples from Spain (207.00 ± 12 μg/L). Note that these apple juice samples showed the highest levels of AOH among all recorded beverages. Among cereals, barley from Argentina showed the highest contamination levels. Nine records of AOH levels in wheat are included in Table 1, the highest AOH average level is in samples collected from Slovenia (39.00 ± 1 µg/Kg), and the lowest is in samples evaluated in Canada with an average level of 2.20 ± 3.3 µg/Kg.

Although AOH is an emerging mycotoxin with no regulated levels in food, the EU set recommendations for its levels in some food categories. In this study, the levels of AOH were recorded in 18 commodities belonging to the EU-indicated food categories, among which only four have levels exceeding the recommended levels (22.2%) (Table 2).

**Table 2:** Levels of AOH in the commodities recorded in this study compared to the EU indicative levels

Type of food	EU indicative levels	*N*^a^	*N*^b^	Country of origin of commodities with AOH exceeding level (product)
Cereal-based foods for infants and young children	2 µg/Kg	1	0	–
Tomato products	10 µg/Kg	14	3	Spain (tomato concentrate) Germany (tomato-based products) Argentina (tomato-based food)
Sunflower oil	10 µg/Kg	0	0	–
Sesame and Sunflower seeds	30 µg/Kg	3	1	South Africa (sunflower seeds)

*N*^a^: number of commodities evaluated in this study under the indicated EU food category

*N*^b^: number of commodities evaluated in this study with AOH average level above the EU indicative level

Considering the toxicity of this mycotoxin and the widespread occurrence of AOH in food products intended for human consumption as shown in Table 1, it is important to have more toxicological studies on other food production stages such as in-field, during transportation and during storage (Escrivá et al. 2017).

## Alternariol-related adverse human health effects

A recent detailed toxigenic profile of AOH and its metabolites using an in silico working model, based on the MetaTox, Swiss ADME, pKCMS, and PASS online computational programs, has confirmed the known cytotoxic, mutagenic, carcinogenic, and endocrine disruptor effects of mycotoxin. The computational model has also predicted other toxicological endpoints for AOH including vascular toxicity, hematotoxicity, diarrhea, and nephrotoxicity (Marin and Taranu 2023). Alternariol has a potential influence on immune system response. Suppressing the pro-inflammatory responses in human epithelial cells and in human macrophages has been described in the literature (Aichinger et al. 2021). In addition, *Alternaria* toxins have a direct effect on the gut microbiome, by affecting the viability of certain strains that usually colonize the gut and play a crucial role in the function of the digestive system (Aichinger et al. 2021).

The chemical structure of AOH has similarities with natural and synthetic estrogen, which suggest an endocrine disruptive role of AOH. Lehmann et al. (2006) were the first to describe the effect of AOH on endocrine pathways by binding it to and activating estrogen receptors (ER). Recent studies showed that the endocrine activity of AOH and its conjugated forms is more complex than previously described. One of the recent findings is the action of AOH as an androgen receptor agonist (Aichinger et al. 2021). More controversially, a recent study shows that *Alternaria* culture extracts have an anti-estrogenic activity. This toxicological effect contradicts the estrogen-mimic effect shown by AOH; however, this can be explained by the ability of perylene quinones compounds within *Alternaria* mycotoxins to interact with aryl hydrocarbon receptor (AhR), which is a key regulator of phase I xenobiotic metabolism. This interaction might degrade ERs or at least modify ER-related signaling (Aichinger et al. 2019).

## Animal model studies evaluating adverse AOH effects

The toxicity of AOH has been explored using animal models such as mice, rats and zebrafish. The exposure route used was mainly through ingestion by adding the mycotoxin to the food or water of the animals. Table 3 summarizes the results of animal model studies in the last two years.

**Table 3:** Animal models experiments results showing AOH health adverse effects in the last two years

Animal Model	Exposure route	Dose range	Dose causing adverse effect	Exposure duration	Adverse effects	End point	References
Swiss albino mice	Oral	600, 800, 1000, and 2000 mg/kg	600 mg/kg	Acute/3 days	–	Death	(Li et al. 2022)
Swiss albino mice	Oral	50, 100, 200, and 400 mg/kg	400 mg/kg	Sub-acute toxicity/28-days	Pulmonary fibrosis and granuloma in lungs Reduced body weight Reduced food and water consumption	Sublethal deformities Death	(Li et al. 2022)
Balb/c mice	Oral	600, 800, 1000, and 2000 mg/kg	600 mg/kg	Acute/3 days	–	Death	(Xin and Shang 2022)
Balb/c mice	Oral	50, 100, 200, and 400 mg/kg	400 mg/kg	Sub-acute toxicity/28 days	Reduced body weight, reduced food and water consumption, and histopathological alterations in vital organs	–	(Xin and Shang 2022)
AB zebrafish	Embryo (4–8 cell stage)	2.5, 5.0, 10, 15, and 20 mg/l	10 mg/l (38.7 µM)	Acute/48 h	–	Death Sublethal deformities	(Fliszár-Nyúl et al. 2022)
AB zebrafish			10 mg/l (38.7 µM)	120 h post-fertilization	Delayed hatching, tail malformation, uninflated bladder, edemas, and curved body axis	Sublethal deformities	(Fliszár-Nyúl et al. 2022)
Sprague–Dawley rats	Oral	22.05 µg/kg	High dose	Sub-acute toxicity/28 days	Histopathological lesions in kidney and liver	Genotoxicity	(Miao et al. 2022)
Sprague–Dawley rats	Oral	Low dose = 5.51 µg/kg Medium dose = 11.03 µg/kg High dose = 22.05 µg/kg	High, low, and medium doses	Sub-acute toxicity/28-days	Mild inflammatory cell infiltration, and edema steatosis	–	(Miao et al. 2022)

It is important to highlight the study conducted by EFSA on AOH toxicokinetic. Oral application of 2000 mg/kg of AOH to NMRI mice showed low absorption of the mycotoxin, as 90% of the admitted dose was recovered from feces, while only 0.06% was encountered in blood. However, it should be noted that a possible digestive tract inflammation might increase absorption and lead to higher toxicity (Schuchardt et al. 2014).

## Alternariol cytotoxicity mechanisms

Toxicological data about AOH are limited, with a lack of good bioavailability and long-term clinical studies. Little is known about exact toxicity mechanisms, bioavailability and stability of AOH in the digestive system. However, *Alternaria* mycotoxins in general have been proven to cause adverse health effects in animals, including cytotoxicity, fetotoxicity and teratogenicity. They are also mutagenic, clastogenic, and estrogenic in microbial and mammalian cell systems and tumorigenic in rats (Escrivá et al. 2017). Among the suggested toxicity mechanisms of *Alternaria* toxins is their ability to alter cell membrane fluidity in intestinal cells, which directly affect the function of the gastrointestinal track (Aichinger et al. 2021). The occurrence of *Alternaria* mycotoxins has been correlated with esophageal cancer, although other mycotoxins were coexisting in countries with high incidence of esophageal cancer (Solhaug et al. 2016).

Many studies have evaluated the effect of AOH on cells. The accumulative data of cell toxicity clearly suggests that this mycotoxin has adverse effects on various cells as summarized in Table 4. In addition to the individual effects of AOH on cells, some studies have shown the synergic effects of AOH with other mycotoxins, the effect of specific ratios of the two *Alternaria* mycotoxins AOH and ATX on cells lines HepG2, HT29, and HCEC-1CT has additional cytotoxicity compared to the effect of each of the mycotoxins individually (Vejdovszky et al. 2017).

**Table 4:** Cytotoxicity of AOH on various cell lines as per the last two-year studies

Cell line	AOH dose	Exposure duration	End points	Mechanisms involved	References
GES-1 human gastric epithelial cell line	2.5, 5, 10, 15, 20, 30 μM	Acute/24 h, 48 h & 72 h	IC50 = 14.84 μM Cell death reached 57.2% of the cells at 30μM	Apoptosis, activation of caspase-3 cleavage, activation of DNA damage pathways	(Lin et al. 2023)
PCS-200–014 Oral epithelial cells	31.25, 62.5, 125, 250, and 500 µg/mL	Acute/24 h	Significant reduction in cells viability starting at 62.50 µg/ml	Dose-dependent cell death	(Ismail et al. 2023)
WI-38 Lung Fibroblast cells	31.25, 62.5, 125, 250, and 500 µg/ml	Acute/24 h	Significant reduction in cells viability starting at 62.50 µg/ml	Dose-dependent cell death	(Ismail et al. 2023)
PNT1A human prostate epithelial cell line	0.001, 0.01, 0.1, 1, 10, 30, and 50 µM	Acute/24 and 48 h	Significant reduction in cells viability starting above 30 µM	Dose-dependent cell death, cell cycle modulation, inflammation, apoptosis, and steroid hormones modulation	(Urbanek et al. 2023)
PC3 prostate adenocarcinoma cell line	0.001, 0.01, 0.1, 1, 10, 30, and 50 µM	Acute/24 and 48 h	Significant reduction in cells viability starting above 30 µM	Dose-dependent cell death, cell cycle modulation, inflammation, apoptosis, and steroid hormones modulation	(Urbanek et al. 2023)
GES-1 human gastric mucosal epithelial	4 ppm	Acute/48 h	Significant reduction in cells viability to 82%	–	(Sun et al. 2023)
WBCs Human white blood cells	0, 3.125, 6.25, 12.5, 25 and 50 µg/mL	Acute/72 h	IC100 = 75.0 ± 5.0 µM	Anti-inflammatory, downregulation of TNF-α, and reduction in INF-γ and IL-1β	(Mahana et al. 2023)
Huh7 Human hepatoma-derived	20 μM	Acute/48 h	Weak effect with cell survival rate almost 50%	–	(Xu et al. 2023a, b)
HepG2 Human hepatic cells	0.1, 10, 50, and 100 µM	4 h	Significant reduction in cells viability starting at 50 µM	Dose-dependent cell death	(Crudo et al. 2020)
SKOV3 human ovarian cancer cell line	0.001, 0.01, 0.1, 1, 10, 30, 50, 70, and 100 µM	24, 48 and 72 h	IC50 = 52.68 µM	Apoptosis and oxidative stress	(Kozieł et al. 2023)
HEPG2 Human hepatic cells	12, 25, 50, and 100 μg/ml	72 h	EC50 = 28 μg/ml	DNA breakage	(Mahmoud et al. 2022)
HeLa	10, 30, 50, 70, and 100 μM	24 h	Significant reduction in cells viability starting at 10 μM	Dose-dependent cell death	(Fliszár-Nyúl et al. 2022)
A549 The lung cancer cell line	35, 60, 85, and 110 and 135 μM	24 h	Significant reduction in cells viability starting at 60 μM	Dose-dependent cell death	(Li et al. 2022)
Ishikawa cells	1, 2.5 and 5 μM	48 h	Cells growth was affected at 5 μM	Dose-dependent cell death	(Aichinger et al. 2022)
HepG2 human hepatoma	Different concentrations	48 h	IC50 = 87.66 ± 3.11 μM	Affect cells proliferation	(Ming et al. 2022)
MDA-MB231 human breast cancer cell line	Different concentrations	48 h	IC50 ≥ 100 μM	No effect	(Ming et al. 2022)
HepG2 human hepatoma	0.1, 1, 10, and 100 μM	24 h	Significant reduction in cells viability started at 10 μM	Interference with cells metabolic activity	(Gerdemann et al. 2022)
HCEC-1CT non-transformed human colon epithelial cells	0.01, 0.1, 1, 5, and 10 μM	24 h	Significant effect appeared at 0.1 μM	Reduces fluidity of cellular membrane	(Rebhahn et al. 2022)
HT-29 colon adenocarcinoma	0.01, 0.1, 1, 5, and 10 μM	24 h	Significant effect appeared at 0.1 μM	Reduces fluidity of cellular membrane	(Rebhahn et al. 2022)
HepG2 Human hepatocytes	0.1, 0.25, 1, 5, 10, 20, 60 and 120 µg/mL	Acute/24 h & 48 h	IC50 = 11.68 ± 4.05 µg/mL	Dose dependent cell death	(den Hollander et al. 2022)
Caco-2 Human enterocytes	0.1, 0.25, 1, 5, 10, 20, 60 and 120 µg/mL	Acute/24 h & 48 h	IC50 = 18.71 µg/mL	Dose dependent cell death	(den Hollander et al. 2022)

It can be inferred from the data in Table 4 that AOH has genotoxicity, and that it can damage DNA at multiple levels causing single-stranded DNA breaks (SSB) and double-stranded DNA breaks (DSB) together with DNA oxidative damage. Alternariol genotoxicity was first observed by Pfeiffer et al. (2007) and then further demonstrated by others. Alternariol metabolism is known to lead to the production of catechols and quinones, such reactive metabolites can undergo redox cycling resulting in reactive oxygen species (ROS) generation. They can also covalently bind to DNA to cause damage (Fernández-Blanco et al. 2014). Many of the ROS associated intracellular events have been spotted in cells exposed to AOH. However, the addition of antioxidants does not modify downstream AOH exposure consequences, including cell cycle arrest. This implies initial mechanisms being involved in AOH genotoxicity prior to ROS production (Solhaug et al. 2016).

Topoisomerases are crucial enzymes in DNA replication and translation as they facilitate chromosome untangling. Alternariol has been proven to inhibit topoisomerase enzymes’ function and to stabilize the intermediate covalent topoisomerase-DNA binding. This leads to DSB and therefore to genotoxicity that can lead to cell cycle arrest (Aichinger et al. 2021; Pinto and Patriarca 2017). Downstream, the DNA damage response pathway has been proven to be activated upon cell exposure to AOH. This is mainly p53 activation, which is a major protein that regulates DNA repair, cell cycle arrest, apoptosis, autophagy, and senescence, and an indicator of carcinogenicity (Solhaug et al. 2013). The activation of p53 leads to increased levels of proteins that repair cell damage, including proliferating cell nuclear antigen (PCNA) which increased levels of p21 (Solhaug et al. 2012). Exposure to AOH also increases intracellular levels of cyclin B, which can lead to cell cycle arrest. It activates AMP-activated protein kinase (AMPK) which usually functions as cellular energy sensor and decreases the activation of the mammalian target of rapamycin (mTOR), which usually regulates cell growth and survival. This signaling pathways would lead to cell autophagy and senescence (Solhaug et al. 2014).

Beside genotoxicity, AOH is known to act as an endocrine disruptor by mimicking estrogen and activating androgen receptors. Androgen/estrogen imbalance and inflammation were observed in prostate cancer in a recent study evaluating different doses of AOH on prostate epithelial cells. At a high dose of 10µM, AOH induced oxidative stress, DNA damage and cell cycle arrest. Interestingly, these effects were proven to be partially mediated by the activation of ERβ, indicating the role of estrogen-mimic in cytotoxicity and genotoxicity of AOH (Kowalska et al. 2021).

## Alternariol anticancer activity

Alternariol and/or its derivatives have shown potential anticancer effects when investigated in a number of preclinical studies. Scientific results indicate that this form of mycotoxin exhibits anticancer effectiveness through several pathways, including cytotoxicity, oxidative stress by ROS, cell cycle arrest, apoptotic cell death, genotoxicity, anti-proliferation, autophagy, and estrogenic mechanisms. All previously discussed AOH toxicity mechanisms may apply to cancer cells, which made scientists explore it as a possible chemotherapy (Islam et al. 2023).

Chemotherapy is a type of anticancer treatment using single or combined chemical components that kill or stop the multiplication and proliferation of cancer cells (Patyal et al. 2023). Due to varied toxicity mechanisms of mycotoxins, those fungal metabolites have recently become the center of attention for scientists working on the development of novel anticancer drugs (de Menezes et al. 2023). Furthermore, mycotoxins are heat-resistant, stable compounds which add to their value as possible anticancer medications (Jafarzadeh et al. 2023).

Among the mechanisms involved in AOH anticancer effectiveness is its cytotoxicity. Cytotoxicity is the first characteristic evaluated in a chemical when considered as an anticancer drug (Anca Oana et al. 2016). Alternariol cytotoxic effect has been demonstrated in many studies. As an example, AOH showed cytotoxic effects on A549 lung cancer cell line and it also improved carcinoma in bulb/c mice models (Li et al. 2022). Alternariol has also been demonstrated to induce oxidative stress in cancer cells. Studies showing AOH ROS generation are numerous. Starting with Bensassi et al (2012), AOH showed a dose-dependent ROS generation, leading to mitochondrial dysfunction-dependent cytotoxic effects in human colon carcinoma (HCT116) cells (Bensassi et al. 2012). Among the anticancer mechanisms, apoptosis is a form of programmed cell death that occurs in human cells, in response to any internal or external cell disturbing event (Fernández-Lázaro et al. 2023). Many anticancer agents are designed to initiate apoptosis in tumor cells. AOH has been demonstrated to induce apoptosis in a mitochondria-dependent pathway, characterized by a p53 activation (Bensassi et al. 2012). Anticancer drugs can also act by exerting genotoxic and mutagenic effects on cancer cells. As previously discussed, AOH is known for its genotoxicity in both normal and cancer cells (Crudo et al. 2023). Anti-proliferative effect is one of the desired mechanisms in an anticancer drug. Previous studies have shown that AOH exerts an anti-proliferative effect in CaCo-2 cells (Vila-Donat et al. 2015). Explored anticancer agents are also studied as autophagy inducers in cancer cells (Kamalzade et al. 2023). A previous study on RAW264.7 macrophage cells showed a dose-dependent increase in autophagy marker LC3 when treated with different concentrations of AOH (Solhaug et al. 2014).

Despite the promising anticancer mechanisms, there are many therapeutic limitations of mycotoxins as anticancer drugs. Limitations include insufficient knowledge of the pharmacokinetics, solubility, and the metabolism of AOH. The main concern in this approach is the insufficient understanding on how AOH would molecularly target tumor cells without causing systematic toxicity to the body (Islam et al. 2023).

## Factors modifying AOH toxicity

Co-infection of some crops such as grains, pome fruits, and grapes with *Alternaria* and other toxigenic strains such *Fusarium*, *Penicillium* and *Aspergillus* is common. Therefore, the co-occurrence of *Alternaria* toxins with other mycotoxins is likely to occur, which makes risk assessment difficult to perform due to the adverse synergic effect that this combination can have on human health (Nan et al. 2022).

Alternariol (AOH) is stable at pH 5 and it can be degraded by 0.18 M phosphate/citrate buffer pH 7 into 6-methylbiphenyl-2,3′,4,5′-tetrol (Siegel et al. 2010). Alternariol also shows stability during pasteurization (Elhariry et al. 2016). Levels of mycotoxins might change during food processing, based on their stability. Surprisingly, clarification of pomegranate juice has been shown to increase AOH levels. This might be due to the presence of conjugated forms of AOH in the juice, which ends up being cleaved into free mycotoxins upon clarification, using proteolytic enzymes (Elhariry et al. 2016).

The application of antioxidants, such as *N*-acetylcysteine (NAC) and ascorbic acid (vitamin C), was not useful in avoiding AOH cell cycle arrest and autophagy effects on cells, which implies initial mechanisms involved in AOH genotoxicity (Chain 2011).

## Alternariol (AOH) spread control

Mycotoxins are concerning natural contaminants that occur in agricultural products and that have adverse human and animal health effects. There is a continuous search for effective prevention measures and control strategies to reduce the levels and therefore the toxicity of these mycotoxins (Awuchi et al. 2021). Strategies to control fungal growth in the first place are among the most effective. However, adverse effects of pesticides on both human health and the environment makes this control controversial (Saleh and Goktepe 2019). Alternatively, scientists are exploring natural products as biological controllers to replace commonly used chemicals. Many studies have shown success in controlling *Alternaria* species in fruits and vegetables using natural oils, plant extracts, bacterial bacteriocins, fungal extracts, algal extracts, and others. Further efforts are to be directed toward the commercialization of these findings (Saleh and Abu-Dieyeh 2021).

Among the successfully described AOH control methods, extrusion showed an AOH level reduction by up to 87%, if processing conditions are optimized (Janić Hajnal et al. 2016). Arginine has also been proven to reduce AOH biosynthesis when applied to fruits at the post-harvest level (Touhami et al. 2016). As AOH has three OH groups in its structure, it can be easily oxidized using cold plasma. However, this method is limited by the low penetration rate of the reactive species responsible for mycotoxins degradation, which keeps its application at the level of superficial food contamination (Ravash et al. 2023). Cold plasma showed a good degradation rate of AOH (up to 60%) in wheat flour samples (Doshi and Šerá 2023). A more sophisticated technique involves dielectric barrier discharge cold plasma, which increases the degradation rate of AOH to 100%, as shown by Wang et al. (2020). Ultraviolet radiation treatment using UVC has shown in some studies a high rate of AOH concentration reduction of up to 80% (Lopes et al. 2023). A recent study has shown the effectiveness of β-cyclodextrin bead polymer (BBP) treatment in reducing AOH levels in red wine (Fliszár-Nyúl et al. 2023). Many *Bacillus species* have been evaluated for their potential as bio-controllers to regulate the growth of fruit and vegetable spoiling agents, and to produce metabolites that can be used in mycotoxins degradation. *Bacillus licheniformis* in particular has shown a high rate of AOH enzymatic degradation by CotA laccase production (Veras et al. 2023). In some cases, food processing steps lead to AOH reduction. For example, dough fermentation for 48 h at 25 °C successfully reduces the level of AOH by 41.5% (Janić Hajnal et al. 2020).

## Conclusion

Anthropogenic activities and global megatrends have affected the geographic distribution of mycotoxin-producing fungi. Globalization has facilitated the introduction of additional fungal strains to new destinations. Global warming has led to increased levels of mycotoxins, including AOH in fields and during storage.

Different detection techniques have been developed to evaluate mycotoxins in food. However, AOH exists in many masked formed, combined with other metabolites. Knowing that masked mycotoxins cannot yet be detected by conventional methods, they can be metabolized back into their native form in the body, add to their risks. Future efforts should focus on the development of detection tools that cover mycotoxins in all their forms.

Food-processing stages are usually not enough to lower the levels of AOH in the final food product. Additional treatments are usually needed, and the literature shows that various techniques have been successfully described to control AOH in food products. However, the consideration of this mycotoxin within the emerging mycotoxins and the lack of studies that focus on the detection of masked forms of mycotoxins is leading to a wider spread of AOH around the world.

As an emerging mycotoxin, levels of AOH in food are not yet regulated. However, the literature shows that exposure levels can reach between 3.8 and 71.6 ng/Kg bw/day, which is above the threshold for the toxicological effect of potential genotoxic substances at 2.5 ng/Kg bw/day. The groups who are most at risk of AOH exposure are those who consume large quantities of fruits and vegetables; notably cereal-based foods, and tomato-based products. Although tolerable levels of AOH have not yet been set, the application of a threshold of toxicological concern (TTC) approach by EFSA indicates a concern when it comes to human exposure to AOH. A ten-year surveillance table was developed in this review to summarize the reported occurrences of AOH in food products around the world. Such surveillances are crucial in raising awareness and in supporting health risk assessors. The data show TTC exceeding levels in four studies conducted on samples from Spain, Germany, Argentina, and South Africa.

Exposure of animal models to AOH showed adverse health effects, which have led to death at higher doses. Cytotoxicity of AOH has been widely evaluated and latest literature gathered in this review shows genotoxicity by direct combination with DNA, causing single-stranded DNA breaks (SSB) and double-stranded DNA breaks (DSB). Proven cytotoxicity mechanisms include the generation of reactive oxygen species (ROS) in cells exposed to AOH. Some studies have explored the usage of AOH as an anticancer treatment to induce apoptosis and autophagy of cancer cells. However, targeting only tumor cells is the main therapeutic limitation of this approach.

The consistency of the evidence collected and the findings of studies have proven that AOH exposure is cytotoxic, carcinogenic and has endocrine disruptor effects. Therefore, the levels of AOH in food products and its risks on human health, require further attention, especially among the populations at risk. It is important to get protected from such a widespread occurring toxicant associated with a range of agricultural and food-based products relevant to human diet. The use of the information presented in this review will lead to a better understanding of AOH as a toxicant. The analysis of the occurrence data gathered will give future health risk assessors solid results that can either be used in recommending further occurrence surveillances or used to set exposure levels and maximum tolerable level of AOH in the near future. This will lead to the application of the most effective preventive measures to protect humans from any possible adverse effects.
